# Importance of latrine communication in European rabbits shifts along a rural–to–urban gradient

**DOI:** 10.1186/s12898-016-0083-y

**Published:** 2016-06-14

**Authors:** Madlen Ziege, David Bierbach, Svenja Bischoff, Anna-Lena Brandt, Mareike Brix, Bastian Greshake, Stefan Merker, Sandra Wenninger, Torsten Wronski, Martin Plath

**Affiliations:** Department of Ecology and Evolution, Goethe University Frankfurt, Max-von-Laue-Str. 13, 60439 Frankfurt am Main, Germany; Department of Biology and Ecology of Fishes, Leibniz-Institute of Freshwater Ecology and Inland Fisheries, Müggelseedamm 310, 12587 Berlin, Germany; Department for Applied Bioinformatics, Goethe University Frankfurt, Max-von-Laue-Str. 13, 60439 Frankfurt am Main, Germany; Department of Zoology, State Museum of Natural History Stuttgart, Rosenstein 1, 70191 Stuttgart, Germany; Bristol Zoological Society, Conservation Science, Clifton, Bristol, BS8 3HA UK; College of Animal Science and Technology, Northwest A&F University, Yangling, 712100 Shaanxi China

**Keywords:** Chemical communication, Communication center, Core marking, Localized defecation, Urban ecology

## Abstract

**Background:**

Information transfer in mammalian communication networks is often based on the deposition of excreta in latrines. Depending on the intended receiver(s), latrines are either formed at territorial boundaries (*between*-*group communication*) or in core areas of home ranges (*within*-*group communication*). The relative importance of both types of marking behavior should depend, amongst other factors, on population densities and social group sizes, which tend to differ between urban and rural wildlife populations. Our study is the first to assess (direct and indirect) anthropogenic influences on mammalian latrine-based communication networks along a rural-to-urban gradient in European rabbits (*Oryctolagus cuniculus*) living in urban, suburban and rural areas in and around Frankfurt am Main (Germany).

**Results:**

The proportion of latrines located in close proximity to the burrow was higher at rural study sites compared to urban and suburban ones. At rural sites, we found the largest latrines and highest latrine densities close to the burrow, suggesting that core marking prevailed. By contrast, latrine dimensions and densities increased with increasing distance from the burrow in urban and suburban populations, suggesting a higher importance of peripheral marking.

**Conclusions:**

Increased population densities, but smaller social group sizes in urban rabbit populations may lead to an increased importance of *between*-*group communication* and thus, favor peripheral over core marking. Our study provides novel insights into the manifold ways by which man-made habitat alterations along a rural-to-urban gradient directly and indirectly affect wildlife populations, including latrine-based communication networks.

## Background

### Mammalian communication through localized defecation sites

The transmission of information in localized defecation sites (latrines) plays a central role in mammalian communication ([1–3], reviewed in [4]). Latrines deposited along territory boundaries are known to serve as a visual and olfactory fence, not only to indicate territorial occupancy, but also to signal the competitive ability of the territory owner(s), e.g., towards neighboring territory holders (*between*-*group communication*; seen in European badgers, *Meles meles* [5, 6]; lemurs [7]; meerkats, *Suricata suricatta* [8], and bushbuck, *Tragelaphus scriptus* [9]). Besides this peripheral marking behavior, several species also establish latrines in central parts of their home ranges—termed core marking—in order to support the monopolization of key resources, such as food, shelter, burrows, or nest sites (seen in European badgers [6, 10], lemurs [4, 7], and Arabian gazelles, *Gazella arabica* [11, 12]). Furthermore, latrines that are located in core areas of home ranges facilitate information exchange between the members of the same social group and thus, can enhance and maintain social bonds or dominance hierarchies (*within*-*group communication* [6, 13, 14]).

### Relative importance of core vs. peripheral marking behavior

Dröscher and Kappeler [4] recently highlighted that we still have a limited understanding about how different ecological factors influence the structure and complexity of mammalian latrine-based communication networks. The relative importance of core vs. peripheral marking behavior seems to depend on population ecological variables; e.g., higher population densities increase competition for territorial space and thus, the necessity to indicate territorial occupancy. This, in turn, favors peripheral over core marking, as suggested for high density rural European badger populations [15, 16] (for European rabbits, *Oryctolagus cuniculus*, see also [17]).

Furthermore, economic considerations predict that the establishment, use, and maintenance of latrines depends on the time and energy animals can effectively invest in their marking behavior [3, 18]. If territory dimensions exceed a certain size, peripheral marking is likely to be replaced by the less time-consuming core marking behavior [3, 4, 18]. Likewise, if the number of individuals that contribute to peripheral marking is low and/or animals need to allocate a considerable proportion of their time to other behaviors—e.g., because they spend more time avoiding predators or human disturbance—latrine distribution patterns should become less complex, and a shift towards core marking would be predicted.

### Effects of urbanization on latrine-based communication networks

Population densities of some mammalian species are higher in urban habitats compared to rural areas ([19–21], reviewed in [22]). Moreover, changes in population densities can be accompanied by differences in social organization, such as smaller social group sizes (European rabbits: [23]) or a less coherent social organization in urban and suburban populations (European badgers: [24–26]). Typical behavioral changes in some urban populations include a reduction in time spent foraging [27] and reduced territorial behavior [24–26], along with smaller territory dimensions (e.g., in raccoons, *Procyon lotor* [27]; European badgers [26]; or red foxes, *Vulpes vulpes* [28]; reviewed in [29]). While the aforementioned species are crepuscular and avoid human disturbance [5, 30], other species, like European rabbits, show extended activity rhythms and reduced anti-predator behavior in urban regions [31, 32], and so they are also unlikely to reduce territorial behavior.

Empirical studies considering the question of how urbanization affects latrine-based communication networks are largely restricted to European badgers [25, 26]. In rural areas, where badgers reached high population densities, both core—(“hinterland marking” [5, 6, 10]) and peripheral marking behaviors were reported, but peripheral marking prevailed [15, 16]. Specifically, peripheral latrines were larger, more densely packed, and showed higher utilization frequencies [16]. By contrast, no peripheral latrines were found in a low-density suburban badger population in Bristol [25] and a high-density urban population in Brighton [26]. In case of the Bristol population, latrines accumulated close to the burrow, suggesting a role of latrines for communication within groups. A recent study by Domínguez-Cebrían and de Miguel [33] investigated the latrine-based communication network of a European rabbit population in a suburban forest of Madrid. Latrines deposited at the territorial periphery were previously hypothesized to signal territory occupancy in rabbits, whereas latrines situated in proximity to the burrow likely facilitate information exchange among group members [13, 14, 34–38]. Domínguez-Cebrían and de Miguel [33] found numbers of latrines to decrease with increasing distance from the burrow system and discuss that rabbits could face a higher predation risk when using peripheral latrines. However, no information was provided by the authors on population densities or social group sizes that would have allowed conclusions regarding the question of how (direct and indirect) effects of urbanization influence latrine-based communication networks in their study population.

### Objectives of this study

European rabbits exchange information about individuals’ age, sex, reproductive condition, and social status via secretions emanating from the anal and submandibular glands [14, 38, 39]. Rabbits deposit hard fecal pellets at latrines that are covered with anal gland secretions [36, 40] and smear secretions from the submandibular gland onto fecal pellets during so-called “chinning” behavior [14, 37, 39, 40]. It is thus well conceivable that latrines at territorial boundaries provide information about territorial occupancy to potential territory intruders (*between*-*group communication*) (e.g., [13, 14, 34–38]). In contrast, the common use of latrines located at core areas by different members of the same social rabbit group is probably mainly related to the establishment and maintenance of social group structures (*within*-*group communication*) [13, 14]. Previous studies were suggestive of a pattern in which peripheral marking is pronounced when population densities are high and distinct social groups are competing ([17], see also [15, 16] for European badger populations).

Population densities of European rabbits in rural areas of Europe are currently on decline [31, 41–44], while at the same time rabbits can reach high densities in urban and suburban areas (for Germany see [31, 43]) but tend to form much smaller social groups [23]. This trend is probably largely caused by intensified agricultural practices in rural areas, where the availability, e.g., of thickets for burrow construction is decreasing [23, 41–44]. Hence, European rabbits are an interesting species to compare population differences in latrine-based communication networks along a rural-to-urban gradient. The paucity of studies investigating the relative importance of core marking (*within*-*group communication*) vs. peripheral marking (*between*-*group communication*) in mammalian latrine-based communication networks further motivated our present study. We investigated rabbit populations along a rural-to-urban gradient. We located latrines at each site and established the distance of each latrine to the nearest burrow. We also assessed latrine dimensions and densities as indicators for long-term use, and numbers of fresh fecal pellets as an indicator for recent use. We further quantified direct and indirect anthropogenic impact at our study sites, including several (interrelated) variables describing human nuisance and anthropogenic landscape alterations (see ‘degree of urbanity’ [23, 31]). This allowed us to establish distribution patterns of latrines relative to the burrow, whereby a prevalence of *core marking* should be reflected by highest latrine densities, larger latrine dimensions, and more fecal pellets per latrine, close to the burrow compared to latrines afar from it. If *peripheral marking* prevails, this should lead to the opposite pattern.

Our predictions were derived from the observation that population densities of rabbits increase, while at the same time social group sizes decrease, along the rural-to-urban gradient considered here [23, 31]. We predicted that *peripheral marking* for territorial defense becomes more important in urbanized regions, as increasing population densities increase competition for space and other resources. Moreover, small group sizes at urban study sites should also favor peripheral over core marking behavior as the necessity to communicate within groups decreases. This should lead to a pattern where latrine densities, sizes, and utilization frequencies increase with increasing distance from the burrow towards the inner parts of the city, while the opposite pattern can be predicted for rural sites.

## Methods

### Selection of study sites

**Fig. 1:**
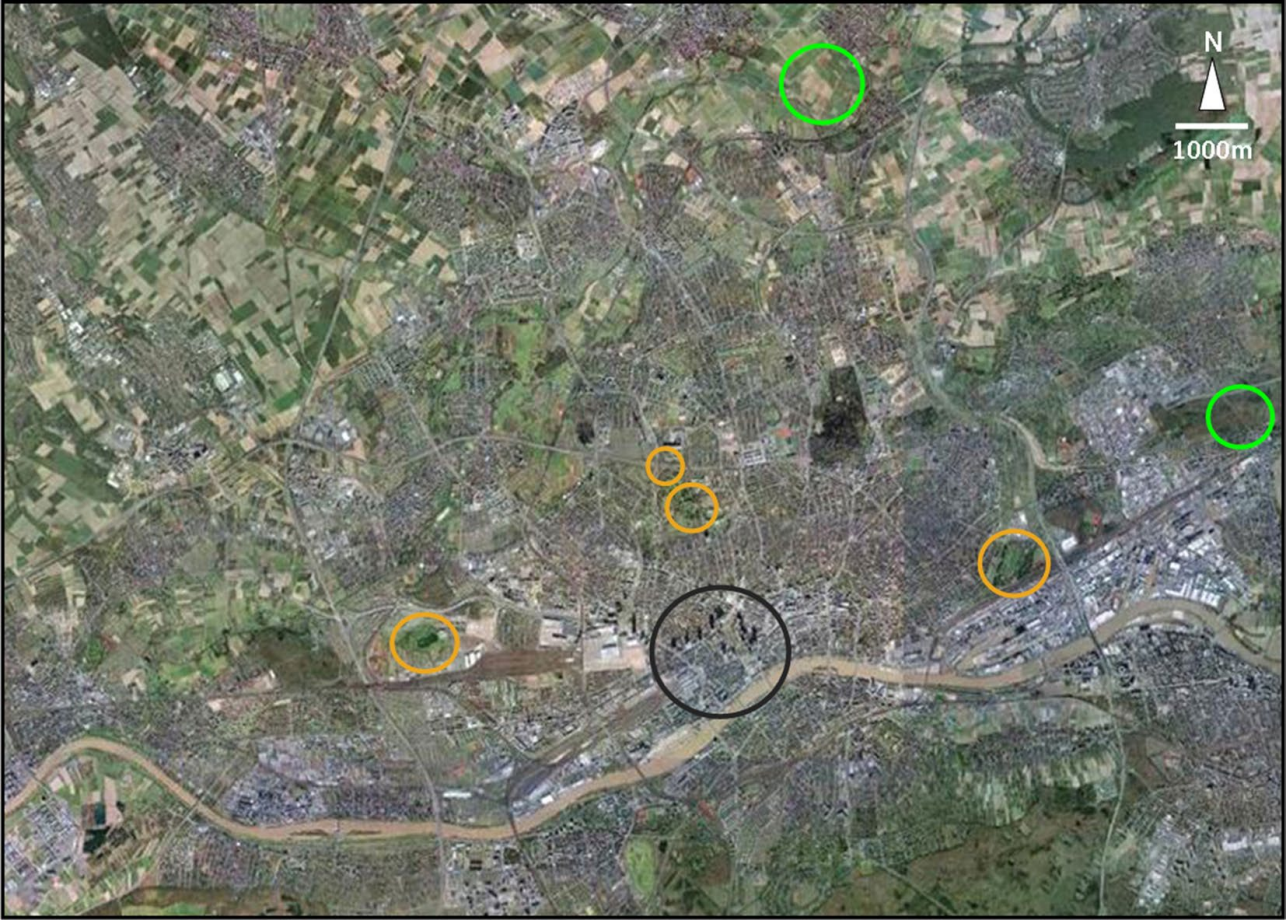
Overview and location of study sites. Locations of all 15 study sites along the rural-to-urban gradient in and around Frankfurt a.M. *Black circles* *n* = 9 urban study sites, *orange circles* *n* = 4 suburban study sites, *green circles* *n* = 2 rural study sites *Source* Google Earth

**Fig. 2:**
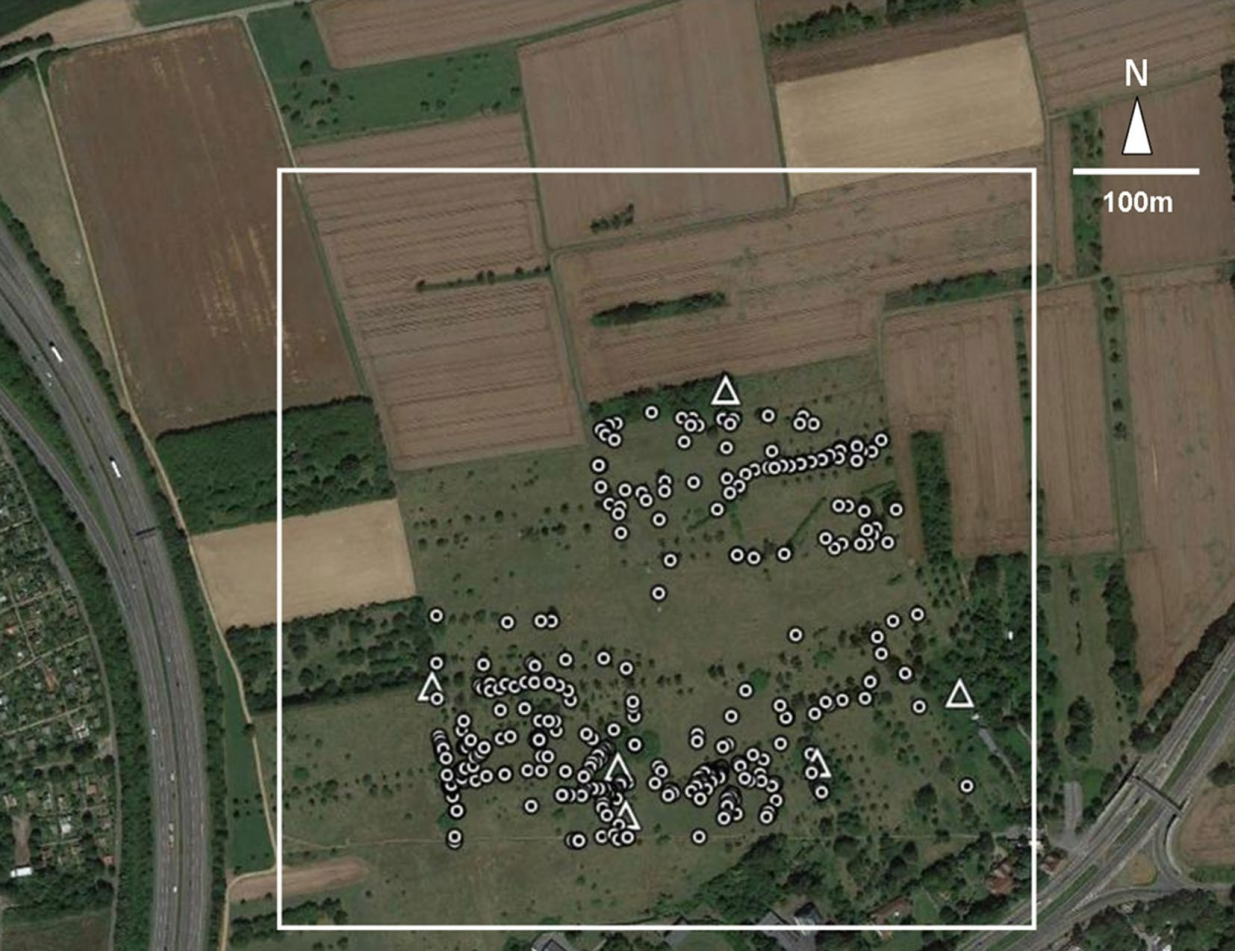
Example of latrine distribution patterns. Detailed aerial photograph of the study site Bad Vilbel. *White triangles* indicate rabbit burrows, *white dots* indicate rabbit latrines *Source* Google Earth

We studied rabbit populations in nine green spaces (measuring between 1 and 4.9 ha in size) in the city center of Frankfurt a.M. (Germany) that are highly fragmented and separated from each other by heavily used roads, in four parks at the periphery of the city (between 5.5 and 30.2 ha) and  at two nearby rural study sites (both 36 ha; Table 1; Figs. 1, 2). Unfortunately, we were not able to include more study sites within the rural surrounding of Frankfurt a.M. due to difficulties in finding areas where a representative population density is still existent.

**Table 1:** Study sites

Study sites	Coordinates		Size [ha]	Degree of urbanity	Population density (rabbits/ha)	Mean social group size
Rural						
Bad Vilbel	N 50°9.418	E 8°41.820	36.00	−2.55	0.88	8.80
Maintal	N 50°8.653	E 8°49.094	36.00	−1.80	3.38	10.00
Suburban						
Ostpark	N 50°7.251	E 8°43.364	30.20	−0.45	19.14	9.50
Grüneburgpark	N 50°7.647	E 8°39.608	27.00	−0.43	0.26	3.50
Rebstockpark	N 50°6.674	E 8°36.773	21.10	−0.36	15.02	4.00
Miquelanlage	N 50°7.970	E 8°39.524	5.50	−0.04	2.27	2.83
Urban						
Site 1	N 50°6.999	E 8°41.503	4.90	0.47	8.16	2.90
Site 2	N 50°6.673	E 8°41.608	3.53	0.47	4.53	4.00
Site 3	N 50°6.723	E 8°40.220	3.64	0.50	9.07	4.00
Site 4	N 50°7.098	E 8°40.946	3.37	0.57	13.95	2.00
Site 5	N 50°7.160	E 8°41.198	2.18	0.59	15.60	3.40
Site 6	N 50°7.001	E 8°40.529	3.66	0.59	3.55	2.00
Site 7	N 50°6.865	E 8°40.263	1.33	0.76	9.02	1.50
Site 8	N 50°6.870	E 8°41.650	1.50	0.84	24.67	1.67
Site 9	N 50°6.606	E 8°40.323	1.00	0.85	5.00	2.00

Detail information for the 15 study sites situated along the rural-to-urban gradient in and around Frankfurt a.M., Germany

In case of the suburban and urban study sites, short-cut meadows were the dominant landscape element (with a grass cutting regime of up to once a week during summer), and the dimensions of our study sites were clearly defined by park borders like streets or pathways. As comparable structures were lacking at both rural sites, we decided to selected quadrants of 600 × 600 m as our study sites, which were sufficiently large to include the outermost latrines afar from the burrow systems (Fig. 2). Here, open landscapes were dominated by agriculturally used areas where meadows (with a sheep grazing regime of two times per year), rape and wheat fields alternated. Between the meadows and fields, only few patches of thickets were present, mainly comprising blackberry bushes (Fig. 2).

### Survey of latrine-based communication networks

We systematically mapped latrines and burrows by two persons walking line transects (app. 5 m apart) across the entire study area within all of our 15 study sites, starting in the early morning. We took GPS coordinates from the center of 3253 latrines and the center of 182 burrow systems using a Garmin 12 GPS [separate burrow systems were identified with the help of local hunters that use domesticated ferrets (*Mustelo putorius furo*) to chase rabbits out of the burrow within the framework of a regular hunting scheme, organized by the city of Frankfurt, hunting licence ID 1000250221]. We collected data during the reproductive season of rabbits, which in our latitude lasts from March to September, when territorial defense is strongest [36, 38]. Urban and suburban study sites as well as the rural study site Bad Vilbel were simultaneously sampled between May and September 2011, while the second rural study site (Maintal) was sampled between June and July 2012. Latrines were defined as an accumulation of at least 20 single fecal pellets within an area of 20 × 30 cm [44]. Based on the GPS coordinates we calculated distances of latrines to the nearest burrow system (see also [33, 35, 45]). We measured several variables for each latrine that are—according to previous studies on mammals, including European rabbits—suitable to characterize latrine-based communication networks [4, 6, 9, 11, 12, 33, 36]. Later we evaluated how those variables change with increasing distance of latrines from the respective burrow system (core vs. peripheral marking, see Statistical analyses). For example, if core marking prevails, latrines close to the burrow should be used more often by the members of the social group than peripheral ones, and this should be reflected by higher numbers of (fresh) fecal pellets compared to latrines that are less often used.

We excluded *n* = 10 burrow systems with less than three latrines from our statistical analyses as those burrows did not show signs of regular use. Moreover, by doing so, we followed the methodological approach of another recent study on latrine distribution patterns of European rabbits in a suburban area [33] so that we were able to discuss our results in comparison to that study.

#### (a) Indicators of long-term latrine use

As one indicator of long-term latrine use, we established latrine sizes by measuring the maximum width and length of the area that fell into our definition of a latrine (see above). We approximated latrine dimensions [m^2^] using a rectangular formula. We also determined numbers of fecal pellets per latrine as another estimate of latrine size. Accurately counting fecal pellets in all latrines through total clearing would have caused an enormous work load, and so we decided to estimate numbers of fecal pellets by eye (see [36]). This estimation method had been practiced before data collection at sites outside of our study area and was confirmed through total clearing after the test trials. As latrine sizes and numbers of fecal pellets both describe latrine dimensions, we log-transformed and subjected both to a factor reduction (principal component analysis, PCA). We retrieved a single PC with an Eigenvalue >1 (1.50) that explained 75.3 % of the total variance, henceforth referred to as ‘latrine dimension’.

Another variable that was used in previous studies to describe the relative importance of core vs. peripheral marking was the latrine density (e.g., latrines were more densely packed at the territorial periphery in a high-density urban badger population [16]). We expressed latrine densities by calculating the mean distance of each latrine to the nearest two neighboring latrines [11, 12].

#### (b) Indicator of recent latrine use

As an indicator of recent latrine use, we noted whether fresh fecal pellets were present (‘0’ no fresh fecal pellets present, ‘1’ fresh fecal pellets present) and if present, we accurately counted them once during the process of latrine mapping in the early morning (see [36]).

#### (c) Indicator of territorial behavior at latrines

We noted whether rabbit paw-scrapings—signs of male territorial behavior [46, 47]—were present at latrines (‘0’ no paw-scrapings present, ‘1’ paw-scrapings present). However, we were unable to accurately quantify actual numbers of paw-scrapings.

#### (d) Effect of woody vegetation on latrine distribution

Finally, we also determined the distance of each latrine to the next woody vegetation (either shrubs or a tree), as this ecological variable is known to affect the placement and utilization frequency of latrines in European rabbits [33, 35].

### Estimating the impact of urbanization

In order to relate (direct and indirect) anthropogenic influences to potential differences in latrine-based communication networks we calculated the ‘degree of urbanity’ for each of our 15 study sites following previous studies [23, 31]. In brief, we assessed the proportion of artificial ground cover (e.g., streets, play grounds) and numbers of anthropogenic objects per ha (e.g., benches, street lamps) at each study site, reflecting the availability of continuous living space. Information on the direct intensity of disturbance by humans (pedestrians and bikers) and leashed or unleashed dogs (per min and per ha) that rabbits were exposed to during their main activity periods at dusk and dawn was obtained through transect counts (for more details see [23, 31]). Additionally, we obtained data on numbers of human residents located within a radius of 500 m from the borders of the study sites from the registration office of Frankfurt a.M. (*Einwohnermeldeamt*, updated: 31.10.2010). These data provide an estimation of overall/peak numbers of visitors in the park areas, as residents tend to walk in nearby city parks.

We subjected the four (log-transformed) variables to PCA. A single principal component was retrieved (henceforth referred to as the PC ‘degree of urbanity’, Table 1) with an Eigenvalue >1 (3.44) that explained 85.9 % of the total variance (Table 2a). For display purpose only, study sites were categorized as rural (‘degree of urbanity’ values ≤ −0.5), suburban (> −0.5 and ≤0.5) and urban (>0.5), while the main statistical analyses were performed using continuous data (see below).

**Table 2:** Degree of urbanity and rabbit population dynamics

	Axis loading
(*a*) Urbanization-related variables	
Proportion of artificial ground cover at each study site	0.84
Numbers of anthropogenic objects per ha at each study site	0.93
Intensity of disturbance by humans and leashed/unleashed dogs min^−1^ ha^−1^	0.97
Numbers of human residents located within a radius of 500 m	0.96
(*b*) Variables related to population dynamics	
Population density	0.89
Burrow density	0.94
Social group size	−0.58

Axis loadings of two separated principal component analyses on variables related to (*a*) urbanization effects (explaining 85.9 % of the total variance) and (*b*) rabbit population dynamics, respectively (explaining 66.7 % of the total variance)

To establish a variable characterizing rabbit population dynamics, we relied on previously published data on rabbit densities (numbers of individuals per ha, assessed by direct census counts along pre-defined transects during dusk and dawn in September/October 2011; Table 1; [31]) and burrow densities [23, 31]. Moreover, we included data on social group sizes, obtained through behavioral observations and augmented by the use of ferrets to drive all members of a social group out of their burrow (Table 1; [23]). Again, we log-transformed the three variables and subjected them to PCA. A single principal component was retrieved with an Eigenvalue >1 (2.00) that explained 66.7 % of the total variance (PC ‘population dynamics’; Table 2b). As both principal components, the ‘degree of urbanity’ and ‘population dynamics’, were highly correlated (Spearman rank correlation: *r* = 0.74, *p* = 0.002, *n* = 15; see also [23, 31]), we decided to include only the ‘degree of urbanity’ in our statistical analyses. Running independent analytical models (see below) with different combinations of both covariates (e.g., ‘population dynamics’ and ‘degree of urbanity’), however, yielded qualitatively very similar results (results not shown).

### Statistical analyses

#### (a) Relative distance of latrines to the nearest burrow (d_rel_)

To compare the spatial distribution of latrines between sites, we first corrected for variation in the sizes of areas marked by latrines around burrow systems, e.g., different home range sizes. Unfortunately, radio-tracking and capture-mark-recapture approaches to establish exact home range dimensions were not feasible for all rabbit groups at our 15 study sites. By using the following approach we were still able to account for variation in home range sizes:

First, based on a distance matrix for all latrines and all burrows at a given study site, each latrine was assigned to the closest burrow (see also [33, 35]). Second, for each burrow we defined the perimeter in which 95 % of all latrines that had been assigned to this burrow were located. Third, we determined the mean distance of the two outermost latrines to the rabbit burrow within this 95 % perimeter (d_max_) and used this value to calculate the dimensions of the latrine-marked area (A [ha]) around each rabbit burrow, assuming the burrow to be the center ($A =\uppi \times d_{{\max}}^{2}$). For every latrine belonging to this burrow system we corrected its absolute distance to the center of the burrow (d_abs_) by d_max_ and thus obtained the relative distance of a latrine as d_rel_ = d_abs_/d_max_. Our approach was justified by the observation that we found latrines that were located close to the respective burrow system and afar from it in all cases, representing cases of core- and peripheral marking (see also [33]). Where we provide descriptive statistics, we categorized latrines depending on d_rel_-values as ≤0.25 (e.g., around the burrow), 0.25–0.50, 0.50–0.75, or ≥0.75 (periphery), while all statistical tests were conducted using continuous data.

In our first approach, we used arcsine (square root)-transformed d_rel_-values as the dependent variable in a linear mixed model (LMM, ‘mixed’ procedure in SPSS 13). We used ‘burrow ID’ as subject-grouping factor with random intercepts specified for each burrow and the ‘degree of urbanity’ as the explaining variable (covariate). A similar approach was used to investigate a potential effect of increasing urbanity on latrine-marked areas around rabbit burrows.

#### (b) Latrine characteristics in relation to the distance to the nearest burrow

In our second approach, we tested whether latrine dimensions and densities, numbers of fresh fecal pellets and distances to the next woody vegetation differed from the core to the periphery of the latrine-marked area, and if this pattern changes along the rural-to-urban gradient. We ran four LMMs using the respective variables (all log-transformed) and again included random intercepts for every burrow system (‘burrow ID’), while ‘d_rel_’-values and the ‘degree of urbanity’ were used as explaining variables (covariates).

We included the interaction term ‘d_rel_ × degree of urbanization’ in the initial model and step-wise removed all non-significant explaining variables from the reduced model starting with the interaction effect. In case of significant interaction terms, we refrained from interpreting main effects and concentrated on the interaction effects. To analyze the binary variables ‘presence of fresh fecal pellets’ and ‘presence of paw-scrapings’ we ran logistic regressions each including ‘d_rel_’, the ‘degree of urbanity’, and their interaction as the explaining variables. Non-significant effects were excluded in a step-wise backwards elimination procedure.

## Results

### Relative distance of latrines to the nearest burrow (d_rel_)

The ‘degree of urbanity’ had a significant effect on mean distances of latrines to the next burrow system (d_rel_; Table 3a), reflecting that distribution patterns of latrines shifted from core- to more periphery-biased along the rural-to-urban gradient. At rural sites, 13.5 ± 0.6 % of all latrines (mean proportion ± SE) were located in the core section close to the burrow (d_rel_ ≤ 0.25) and 25.3 ± 1.6 % at the relative periphery (d_rel_ ≥ 0.75). By contrast, only 3.4 ± 1.1 % of latrines were established within the core section at urban study sites, while 34.6 ± 7.0 % of latrines was found at the periphery of the latrine-marked area. At suburban study sites, 11.7 ± 2.1 % of latrines were located in the core section and 33.2 ± 4.7 % at the periphery.

**Table 3:** Univariate linear mixed models

Fixed effects	*F*	*df*_1_, *df*_2_	*P*
(*a*) d_rel_			
‘Degree of urbanity’	11.13	1, 93	0.001
(*b*) Latrine-marked area (A)			
‘Degree of urbanity’	25.49	1, 126	<0.001
(*c*) Latrine dimension (PC on latrine size and numbers of fecal pellets)			
‘Degree of urbanity’	3.04	1, 531	<0.001
‘d_rel_’	0.29	1, 2960	0.589
‘d_rel_ x degree of urbanity’	5.33	1, 2870	<0.001
(*d*) Latrine density			
‘Degree of urbanity’	10.67	1, 190	0.001
‘d_rel_’	34.74	1, 2953	<0.001
‘d_rel_ x degree of urbanity’	5.26	1, 2900	0.022
(*e*) Numbers of fresh fecal pellets			
‘Degree of urbanity’	0.77	1, 269	0.38
‘d_rel_’	0.91	1, 295	0.34
‘d_rel_ x degree of urbanity’	0.98	1, 521	0.32
(*f*) Distance to next woody vegetation			
'Degree of urbanity'	11.31	1, 2973	0.001
'd_rel_'	354.29	1, 2853	<0.001

Results of univariate LMMs using (*a*) ‘d_rel_’, (*b*) ‘latrine-marked area (A)’, (*c*) ‘latrine dimension’, (*d*) ‘latrine density’, (*e*) ‘numbers of fresh fecal pellets’ and (*f*) ‘distance to next woody vegetation’ as dependent variables

We also detected a significant effect of the ‘degree of urbanity’ on the dimensions of the latrine-marked area around rabbit burrows ('Latrine-marked area'; Table 3b), which decreased from 2.73 ± 0.48 ha at rural sites, over 2.11 ± 0.27 ha at suburban sites, to 0.87 ± 0.25 ha at urban study sites.

### Latrine characteristics in relation to their distance to the nearest burrow

#### (a) Indicators of long-term latrine use

**Fig. 3:**
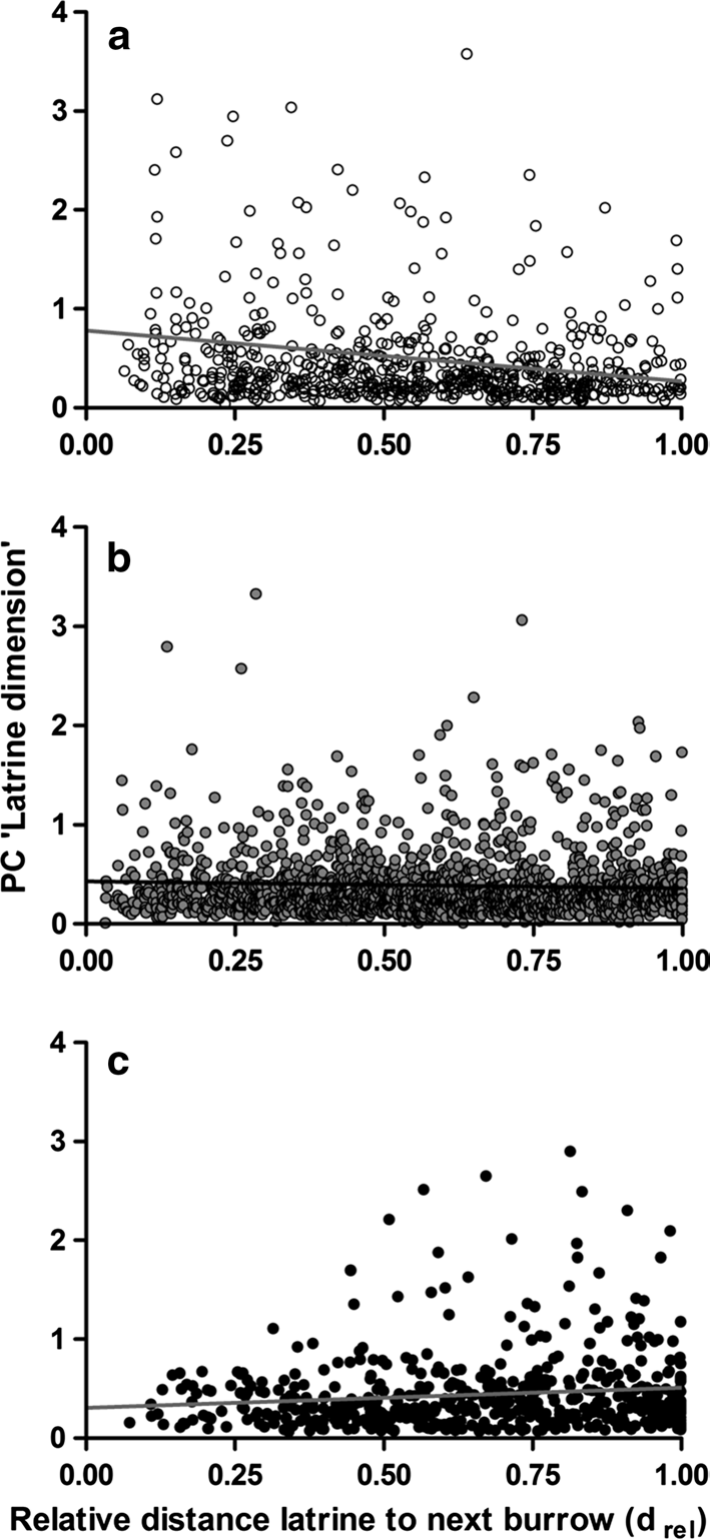
Latrine dimension. Correlation between the PC ‘latrine dimension’ (incorporating the size of latrines [m^2^] and numbers of fecal pellets) and the relative distance to the next burrow (d_rel_) at (**a**) rural sites with ‘degree of urbanity’ values ≤ −0.5 (*n* = 547 latrines), (**b**) suburban sites with ‘degree of urbanity’ values > −0.5 and ≤0.5 (*n* = 1828), and (**c**) urban sites with ‘degree of urbanity’ values >0.5 (*n* = 652 latrines)

Latrine dimensions were affected by the ‘degree of urbanity’ and the interaction term ‘d_rel_ × degree of urbanity’ (‘Latrine dimension’; Table 3c). While latrine dimensions at rural study sites became smaller with increasing distance from the next burrow (Fig. 3a), the opposite pattern was observed at urban study sites: latrines that were located at the relative periphery of the latrine-marked area were larger than those located close to the burrow (Fig. 3c). Regarding suburban sites, latrine sizes showed no notable variation within the latrine-marked area (Fig. 3b).

**Fig. 4:**
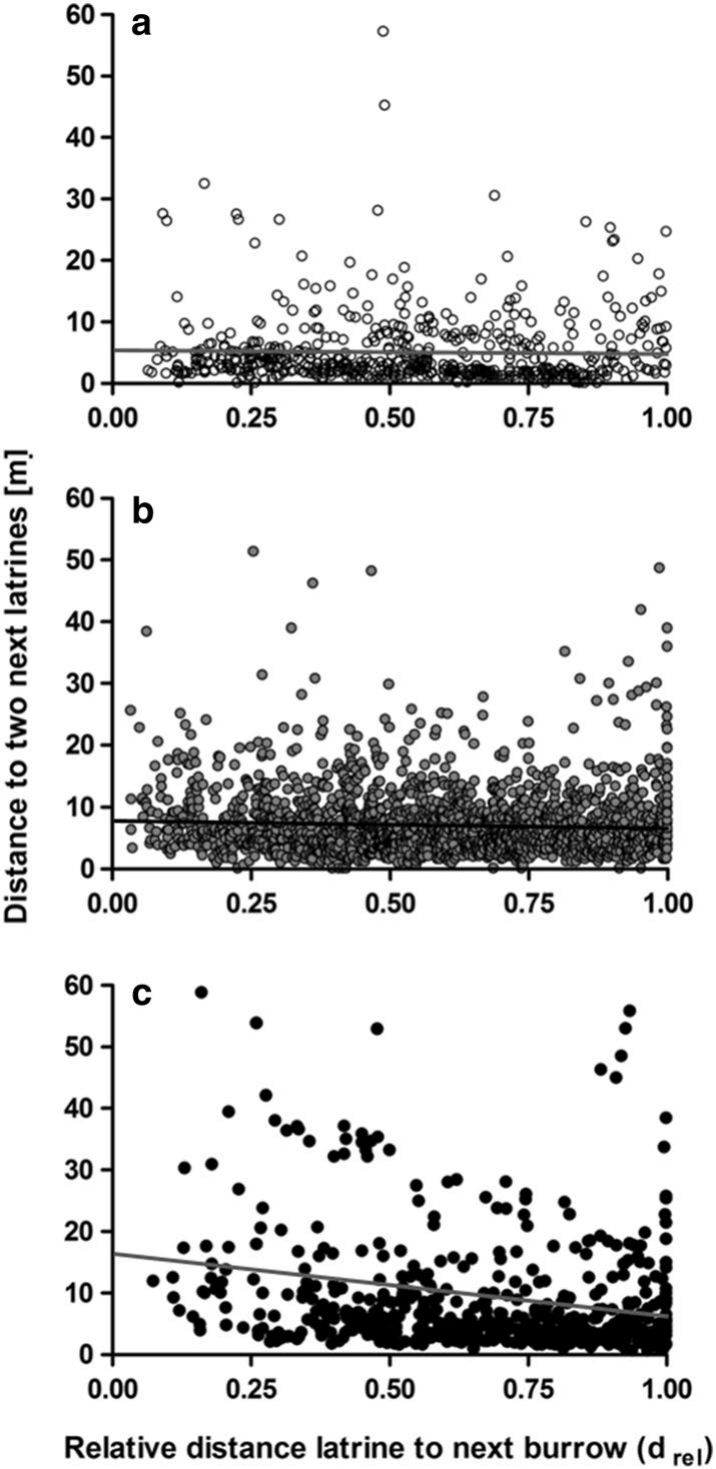
Latrine density. Correlation between latrine density (expressed by the mean distance of a latrine to the nearest two neighboring latrines [m]) and the relative distance to the next burrow (d_rel_) at (**a**) rural sites with ‘degree of urbanity’ values ≤ −0.5 (*n* = 547 latrines), (**b**) suburban sites with ‘degree of urbanity’ values > −0.5 and ≤0.5 (*n* = 1828), and (**c**) urban sites with ‘degree of urbanity’ values >0.5 (*n* = 652 latrines)

Considering latrine densities, the ‘degree of urbanity’, ‘d_rel_’ and the interaction term ‘d_rel_ × degree of urbanity’ had significant effects (‘Latrine density’; Table 3d). The latrine density decreased slightly with increasing distance from the next burrow system at rural study sites (Fig. 4a). By contrast, at urban sites latrine densities were considerably higher at the relative periphery of the latrine-marked area compared to latrines located close to the burrow (Fig. 4c). At suburban study sites, latrine densities did not vary throughout the latrine-marked area (Fig. 4b).

#### (b) Indicator of recent latrine use

As an estimate of the frequency of recent latrine use, we analyzed presence of fresh fecal pellets in each latrine. The logistic regression revealed a negative correlation between the ‘degree of urbanity’ and the presence of fresh fecal pellets within latrines (*B* = −0.17, *Wald* = 13.96, SE = 0.046, *P* < 0.001, −2log likelihood = 2884.71, Nagelkerke *R*^2^ = 0.007; all excluded variables: *P* ≥ 0.29), suggesting that the proportion of latrines that contain fresh fecal pellets decreased along the rural-to-urban gradient. Considering only the subset of latrines that contained fresh fecal pellets, our mixed model revealed no significant relations between the dependent and independent variables (‘Numbers of fresh fecal pellets’; Table 3e).

#### (c) Indicator of territorial behavior at latrines

Regarding the presence of paw-scrapings at latrines the logistic regression uncovered a positive correlation with the ‘degree of urbanity’: the proportion of latrines at which paw-scrapings were present increased along the rural-to-urban gradient (*B* = 0.57, *Wald* = 176.27, SE = 0.043, *P* < 0.001, −2log likelihood = 3637.78, Nagelkerke *R*^2^ = 0.083; all excluded variables: *P* ≥ 0.38). In 76.6 ± 2.0 % of all latrines mapped at urban study sites paw-scrapings were present (mean percent latrines with paw-scrapings present ± SE), while this was only the case in 43.8 ± 1.8 % of all latrines at rural study sites and 70.4 ± 1.1 % of latrines at suburban sites.

#### (d) Effect of woody vegetation on latrine distributions

Finally, the distance of latrines to the next woody vegetation was affected by ‘d_rel_’ and the ‘degree of urbanity’ (‘Distance to next woody vegetation’; Table 3f). The distance between latrines and the next tree or shrub increased with increasing distance from the burrow, reflecting that most burrows were situated in dense vegetation. At core sections (d_rel_ ≤ 0.25), the mean (± SE) distance of latrines to the next woody vegetation was 8.72 ± 0.98 m (*n* = 318), while at the periphery (d_rel_ ≥ 0.75) mean distances were 16.10 ± 0.58 m (*n* = 896). Along the rural-to-urban gradient, the mean distance of latrines to the next woody vegetation was shortest for urban areas (5.66 ± 0.69 m, *n* = 652) compared to rural (14.95 ± 0.65 m, *n* = 547) and suburban sites (16.83 ± 0.38 m, *n* = 1828).

## Discussion

Our present study is the first to demonstrate gradual variation in the relative importance of different latrine marking strategies in European rabbit populations along a rural-to-urban gradient. The results comply with our prediction that higher rabbit population densities in urban regions, along with smaller group sizes (pairs and their offspring, and partly even solitary individuals [23, 31]), bring about an increased necessity for *between*-*group communication*, e.g., to claim territorial occupancy through peripheral marking. Not only were relatively more latrines located at the periphery of the rabbit burrow in urban populations, but those latrines were also larger in size, more densely packed and more frequently used. This trend contrasted with a strong signature of core marking in rural rabbit populations.

Fewer group members contributing to the establishment and maintenance of latrine-based communication networks in urban rabbit populations likely explain why the proportion of latrines with fresh fecal pellets was lower. Moreover, higher ambient temperatures and altered patterns of precipitation and evaporation are typical of urban regions—caused by the high proportion of sealed surfaces [48, 49]—possibly accelerating the decay of fecal pellets. Also, some fecal pellets will be regularly removed during the maintenance of green spaces, which, according to information provided by the *Frankfurter Grünflächenamt*, reaches its maximum in urban parks. Accordingly, using numbers of fecal pellets and fresh fecal pellets, respectively, as dependent variables to characterize latrine-based communication networks in urban, suburban and rural mammalian populations needs to be considered with caution. Likewise, those variables are sometimes used to estimate local rabbit population densities, which can also provide misleading information (see also [50]). Competition for space and other resources in the small and highly fragmented urban parks is probably intense, given that both the proportion of sealed surface areas and population densities were high, while home range areas marked by latrines were small. We argue that strong competition brings about an increased importance of peripheral marking behavior (see also [15–17]). This is also reflected by the fact that more paw-scrapings (which males use for territory demarcation) were found in latrines at urban study sites.

Following Domínguez-Cebrían and de Miguel [33], another important factor that likely affects latrine-based communication networks in rabbits is predation risk [33, 51]. Common predators of European rabbits in Germany can also reach high densities in cities (foxes [20]; mustelids like *Martes foina* and *Mustela erminea* [30]; domestic cats [52]; crows, *Corvus corone* and magpies, *Pica pica* that prey on juvenile rabbits [53]). However, the fact that those species can reach high densities in cities does not necessarily mean that they exert strong predation on urban rabbit populations (“the predation paradox” [54], reviewed in [22]). For example, several studies demonstrated that those predators can use other abundant food sources in cities [22, 55]. Moreover, both, predator and prey species can alter their activity patterns in urban regions, again leading to an altered predator exposure [56]. Unfortunately, we were not able to systematically quantify predation risk at our study sites. Still, decreased flight initiation distances in suburban and urban rabbits [31] and less time spent exhibiting anti-predator behavior [32] suggest that predation of urban and suburban rabbits may indeed be lower compared to rural populations. At rural sites, rabbits that use latrines at the periphery of their home ranges may be more exposed to predators, while reduced predation risk in urban populations leaves more time to establish and maintain complex communication networks involving latrines afar from the burrow.

When considering distances between latrines and the nearest woody vegetation, shorter distances in urban areas likely reflect more heterogeneous landscapes in cities [54, 56]. In contrast, rural study sites were mostly agriculturally used and are characterized by open and homogeneous landscapes with scarce woody vegetation. In line with the interpretation that sufficient shelter (shrubs and trees) eases burrow formation, a previous study found burrows to become more uniformly distributed along the rural-to-urban gradient considered here [23]. Rabbits prefer to establish latrines on bare soil, clearings, or elevated areas, often close to conspicuous landscape elements such as bushes, trees or anthropogenic objects, while avoiding densely vegetated areas [33, 36]. Not only does this increase the visibility and accessibility of latrines, but it could also reduce the risk of falling victim to avian and terrestrial predators during latrine visits [35]. At our rural study sites, most latrines were found on meadows with short grass, especially close to pathways, while crop fields were largely avoided. By contrast, landscape elements appear to not have such a strong effect on latrine distribution patterns at suburban and urban study sites, where meadows with short grass prevailed.

In contrast to European rabbits, groups of European badgers showed no peripheral marking behavior in urban regions—even at the few sites where the home ranges of different groups overlapped [25, 26]. Davison et al. [26] argued that urban badger groups were rather isolated even where population densities were high, reducing the need for territory demarcation (see also [25]). This was clearly not the case in our study, in which distinct social groups of rabbits occupied territories in close proximity to one another at urban and suburban study sites. Furthermore, crepuscular, timid species like badger are less likely to habituate to permanent anthropogenic disturbance compared to European rabbits (see above). Badgers are probably more distracted from latrine marking by human disturbance than rabbits (see also [4]). Moreover, badger home ranges are considerably larger than those of European rabbits (mean 95 % kernel group home range sizes of urban badgers: 4.71 [26] vs. 0.62 ha for suburban and urban European rabbit populations, unpubl. data). This renders peripheral marking in badgers even more challenging under intense anthropogenic disturbance.

## Conclusions

Human activities affect urban wildlife populations, e.g., through anthropogenic nuisance, habitat fragmentation, and altered food availability (reviewed in [22, 29]). Behavioral changes in urban populations compared to populations inhabiting rural areas (like altered flight- or ranging behavior [22, 29]) are often interpreted as a *direct* consequence of animals having to cope with those novel ecological conditions. Our present study demonstrates behavioral changes in European rabbits, namely altered distribution patterns of latrines relative to the corresponding burrow. Based on previous studies on this and other mammalian species, we argue that increased peripheral marking in urban populations reflects an increased importance of *between*-*group communication* (rather than *within*-*group communication*), and this seems to be a consequence of higher population densities, smaller group sizes, and altered predation risk. Our study adds to our knowledge about the function of mammalian latrines as centers for information exchange between individuals, and—more generally—points towards *indirect* effects of anthropogenic landscape alteration and human nuisance on the behavior of urban wildlife populations. If our interpretations are correct, our results have implications for the conservation and management of rabbit populations: while rural rabbit populations suffer from a loss of suitable habitat [23, 31, 41–44], rabbit populations in urban areas might show higher intrinsic mortality rates arising from high intraspecific competition, while suburban habitats may currently provide an advantageous combination of structural heterogeneity and comparatively low levels of competition. Ongoing studies are trying to assess the potential role of cities in the future conservation of this species, e.g., by providing population genetic information on potential source-sink dynamics in population development. Another aspect to be considered in future studies is that urban and suburban rabbit populations may serve as ecosystem engineers; e.g., nutrients accumulate at latrines, which could have implications for local plant communities and possibly seed dispersal [56, 57]. As “fertile islands”, latrines likely further increase habitat heterogeneity in urban and suburban landscapes [57].

## Abbreviations

a.M.: am Main
d_max_: maximum distance of a latrine to the center of the next burrow
d_rel_: relative distance of a latrine to the center of the next burrow
d_abs_: absolute distance of a latrine to the center of the next burrow
